# Supplementary material for: Personality and psychopathological characteristics in functional movement disorders
Source: PLoS One. 2024 May 10;19(5):e0303379. doi: 10.1371/journal.pone.0303379 (PMC11086865; doi:10.1371/journal.pone.0303379)
Supplement: S1 Table — Abbreviations: Adj D: Adjusted Difference Score. CDI: Coping Deficit Index; SumY: Sum of diffuse shading determinants; FC: Form-color; CF: Color-form; C: Color; FM: Animal Movement; SumC’: Sum of color; WSumC (Weighted Sum of Color); SumT: Sum of texture determinants; SumV: Sum of vista determinants; Zd: Efficiency index; XA%: Accuracy in perception; X-%; distortion in perception; P: Popular; DEPI: Depression index; HVI: Hypervigilance Index; Fr: Rf: SumH: Sum human; An: Anatomy; Xy: X-ray; GHR: Good human representational responses; PHR: Poor human representational responses. (DOCX) [file pone.0303379.s002.docx]

**S1 Table. Description and interpretation of the Rorschach variables.**

| **Variables** | **Description** | **Interpretation** |
| --- | --- | --- |
| **Control and stress tolerance** | | |
| Adj D | Control and coping abilities under the best circumstances | Adj D<0: control and stress tolerance likely limited |
| CDI | Ability to cope with social and highly stressful situations | CDI<3: difficulty coping with highly stressful situations |
| SumY | Unintended negative feelings and emotions stress associated | SumY>2: situational stress affecting emotions |
| FC:CF+C | Expression of emotions control: FC, appropriate; C, unrestrained; CF: less restraint than an FC but better control than C | FC>CF+C: good emotional reactions and displays control |
| **Ideation** | | |
| FM | FM: unwanted and out of control peripheral thought experiences, need states | FM<2: low level of peripheral thought; FM>5: high level of peripheral thought |
| SumC’ | Tendency to suppress or inhibit negative emotions | SumC’>2: holded back emotions that the subject would like to display |
| WSumC | Way of emotions releasing (controlled vs. uninhibited) | SumC’>WSumC: more withholding emotions than are typical |
| SumT | Needs for emotional closeness; openness to close relationships | SumT>1: more needs for closeness than are typical |
| SumV | Self-examination | SumV>0: focus on self-negative aspects. Feeling of guilt or shame |
| **Information processing** | | |
| Zd | Environment scanning | Zd<-3: underincorporative style |
| **Cognitive mediation** | | |
| XA% | World perception | XA%<0.7: difficulty in perceiving world accurately |
| X-% | Distortion in blot perception | X-%>0.25: reality distortion; behavior influenced by misperceptions |
| P | Blot perceived in the same way many others did | P>6: Individuality sacrification for the sake of fitting in with others and with social expectations |
| **Affect** | | |
| DEPI | Affect variable | DEPI>5: affective difficulties, including depression and anxiety |
| HVI | Hypervigilance | Hypervigilance, high vulnerability, mistrust others |
| **Self-perception** | | |
| Fr+Rf | Use of reflection or mirror image based on the symmetry of the blot | Fr+Rf>0: possible inflated sense of self-worth |
| **Interpersonal perception** | | |
| SumH | Awareness of and interest in others | SumH<3: low interest in others; SumH>5: high interest in others |
| An+Xy>1 | Anatomy and X-ray imaging | An+Xy>1: more focus on bodily functioning than is typical |
| GHR-PHR | Interpersonal relationship | GHR>PHR: less adaptive and inappropriate interpersonal behaviors |
